# The development of a multidisciplinary fall risk evaluation tool for demented nursing home patients in the Netherlands

**DOI:** 10.1186/1471-2458-6-74

**Published:** 2006-03-21

**Authors:** Jacques CL Neyens, Béatrice PJ Dijcks, Jolanda CM van Haastregt, Luc P de Witte, Wim JA van den Heuvel, Harry FJM Crebolder, Jos MGA Schols

**Affiliations:** 1 Nursing Home de Riethorst, P.O. Box 35, 4931 AA Geertruidenberg, The Netherlands; 2 iRv, Institute for Rehabilitation Research, P.O. Box 192, 6430 AD Hoensbroek, The Netherlands; 3 Department of Health Care Studies, Maastricht University, P.O. Box 616, 6200 MD Maastricht, The Netherlands; 4 Vivre, Polvertorenstraat 4, 6211 LX Maastricht, The Netherlands; 5 Department of General Practice, Maastricht University, P.O. Box 616, 6200 MD Maastricht, The Netherlands; 6 Faculty of Social and Behavioural Sciences, Department Tranzo, Tilburg University, P.O. Box 90153, 5000 LE Tilburg, The Netherlands

## Abstract

**Background:**

Demented nursing home patients are at high risk for falls. Falls and associated injuries can have a considerable influence on the autonomy and quality of life of patients. The prevention of falls among demented patients is therefore an important issue. In order to intervene in an efficient way in this group of patients, it is important to systematically evaluate the fall risk profile of each individual patient so that for each patient tailor-made preventive measures can be taken. Therefore, the objective of the present study is to develop a feasible and evidence based multidisciplinary fall risk evaluation tool to be used for tailoring preventive interventions to the needs of individual demented patients.

**Methods:**

To develop this multidisciplinary fall risk evaluation tool we have chosen to combine scientific evidence on the one hand and experts' opinions on the other hand. Firstly, relevant risk factors for falling in elderly persons were gathered from the literature. Secondly, a group of Dutch experts in the field of falls and fall prevention in the elderly were consulted to judge the suitability of these risk factors for use in a multidisciplinary fall risk evaluation tool for demented nursing home patients. Thirdly, in order to generate a compact list of the most relevant risk factors for falling in demented elderly, all risk factors had to fulfill a set of criteria indicating their relevance for this specific target population. Lastly the final list of risk factors resulting from the above mentioned procedure was presented to the expert group. The members were also asked to give their opinion about the practical use of the tool.

**Results:**

The multidisciplinary fall risk evaluation tool we developed includes the following items: previous falls, use of medication, locomotor functions, and (correct) choice and use of assistive and protective devices. The tool is developed for the multidisciplinary teams of the nursing homes.

**Conclusion:**

This evidence and practice based multidisciplinary fall risk evaluation tool targets the preventive interventions aimed to prevent falls and their negative consequences in demented nursing home patients.

## Background

Falls and fall related injuries are a major problem in community residing elderly persons and even more in frail elderly residing in institutions. Fall incidents occur frequently in nursing homes and may have considerable consequences for the health status and quality of life of the patients involved, especially if the fall results in a hip fracture. In the Netherlands the mean incidence of fractures for psychogeriatric patients in nursing homes is 26.3 to 28.8 per 1000 beds per year [1]. Due to these fractures, not only the nursing care load increases, but also the mortality risk of patients.

Dementia is a major risk factor for falling [2-4]. Demented patients show a gradually deteriorating mobility and a diminishing ability to recognise, judge and avoid hazards. In Dutch nursing homes about 55% of the patients suffer from dementia but they are involved in 75% of the fall incidents [5]. Therefore, it can be concluded that all demented patients in nursing homes are at high risk of falls. This stresses the importance of taking adequate preventive measures to prevent falls in this group of patients.

Research data indicate positive effects of multifactorial interventions targeted at the prevention of fall incidents [6]. This evidence mostly concerns community dwelling people. Despite the magnitude of the problem of fall incidents in (demented) nursing home patients, only limited evidence is available for the effectiveness of fall prevention among these patients [6,11]. Fall risk assessment tools and preventive interventions developed for the general population of elderly persons seem to be inappropriate for demented patients. The present study aims to contribute to the development of a specialised fall preventive intervention for demented nursing home patients, feasible for the nursing home staff. In order to intervene in an efficient way in the group of demented patients (who all can be considered to be at high risk for falls), it is important to systematically evaluate the fall risk profile of each individual patient so that for each patient tailor-made preventive measures can be taken. Therefore, the objective of the study presented in this article is to develop a feasible and evidence based multidisciplinary fall risk evaluation tool to be used for tailoring preventive interventions to the needs of individual demented patients.

## Methods

The development of this multidisciplinary fall risk evaluation tool consisted of the following four steps:

1. Searching the literature for risk factors for falling;

2. First consultation of experts: suitability of factors;

3. Final selection of risk factors;

4. Second consultation of experts: practical use of the tool.

Below the methods used in each step are described.

### Step 1: Searching the literature for risk factors for falling

A search in PubMed, Medline and Cinahl (from January 1986 until July 2002) was performed to collect scientific publications about risk factors for falling. The search strategy used was: [fall(s) AND elderly] AND [nursing home(s) OR long term care OR risk factor(s) OR assessment OR dementia]. The abstracts of the publications found were screened in order to make a first selection of potentially relevant papers. All papers that addressed risk factors for falling in the elderly were included in this first selection, irrelevant of whether they referred to elderly people residing in the community, hospitals or institutions for long term care. The full text of the publications included, were retrieved and the papers were screened for relevant information about risk factors for falling among elderly people (65+). Subsequently a list of risk factors for falls was made. A risk factor was included in the list if a relationship between the factor and falls in the elderly was reported.

### Step 2: First consultation of experts: suitability of factors

A group of national experts (N = 11) in the field of falling, fall prevention, guideline development and implementation was assembled. The members of this group were researchers from the Free University Amsterdam (VU), Maastricht University (UM), the Dutch Organisation for Applied Scientific Research (TNO), representatives of different disciplines working in a nursing home (nursing home physician, nurse, physiotherapist and occupational therapist), a representative of the Dutch Branch Organisation for Nursing Homes (Arcares), and a representative of the Dutch Association of Nursing Home Physicians (NVVA).

In a plenary meeting the large list of risk factors resulting from step 1 was presented to the experts. Each expert was asked to judge for each risk factor whether it seemed relevant to include it in a multidisciplinary fall risk evaluation tool for demented nursing home patients. They were asked to take into account the daily care process in Dutch nursing homes. In the Netherlands it is common to perform a general comprehensive assessment shortly after admission of a patient to the nursing home. The multidisciplinary fall risk evaluation tool should not overlap with this assessment but should be complementary to it.

In order to generate a compact list of the most relevant risk factors for demented nursing home patients, we reduced the list of risk factors resulting from step 1 using the following criterion: during the expert meeting at least 75% of the experts present had to agree on the importance of this factor.

### Step 3: Final selection of risk factors

Subsequently the members of the research team wanted to make a further selection of the factors resulting from step 2, in order to compose a feasible multidisciplinary fall risk evaluation tool. To do this, they developed the following list of criteria:

• the factor has been described as a risk factor for falling in at least one article addressing nursing home care;

• evaluation of the factor among demented nursing home patients has to be possible;

• the factor must be modifiable; and

• appropriate interventions to reduce or eliminate the risk factor among demented nursing home patients (applicable in daily nursing home routine) are or can be made available.

Next the research team assessed whether the factors resulting from the first selection matched these criteria. Because this did not lead to a substantial reduction of the number of factors, the following criterion was added to further reduce the number of factors:

• the reported Odds Ratio/Relative Risk Ratio of the factor has to be 1.5 or higher.

### Step 4: Second consultation of experts: practical use of the tool

The list of factors resulting from step 3 was presented to the participating experts. By means of a structured (e-mail) questionnaire, we asked them to give their opinion regarding aspects of the practical use of the multidisciplinary fall risk evaluation tool. For every risk factor incorporated in the tool, the experts had to judge (yes or no) the proposals of the research team regarding how, by whom and at which moment in the care process it should be assessed. If the majority answered positive the tool became final.

## Results

### Searching the literature for risk factors for falling

Over forty risk factors for falls have been described in the literature regarding elderly people residing in the community, hospitals and institutions for long term care [12-51]. Most studies consider elderly people in the community. Publications about falls and fall related aspects in nursing homes are relatively scarce. Table 1 shows the risk factors that matched the inclusion criteria of our literature search.

### First consultation of experts: suitability of factors

Seven of the eleven experts who were invited to join the expert meeting, actually attended the meeting. Risk factors that were considered to be relevant for a multidisciplinary fall risk evaluation tool among demented nursing home patients, according to at least 6 of the 7 experts present were: previous falls, chronic disease(s), medication, disturbed vision, independency of transfers, disturbed balance, bad quality of co-ordination, mobility impairments, muscle weakness, foot defects, assistive devices, and protective devices.

### Final selection of risk factors

Table 2 shows the results of the final selection procedure of items for the multidisciplinary fall risk evaluation tool. The risk factors that are shown in this table are those that are considered to be relevant by 6 out of 7 experts. Column 1 shows the required expert group consensus score. Column 2 shows if the items in question are mentioned in literature addressing nursing homes. Columns 3 and 4 show respectively the possibility for evaluation in daily nursing home practice and whether the risk factors can be modified. Column 5 shows whether interventions to reduce or eliminate the risk factors (applicable in nursing homes), are or can be made available. Column 6 shows the scores on the additional criterion (OR/RR = 1.5) to further reduce the list. Column 7 shows which factors fulfilled all inclusion criteria. Eight factors fulfilled all criteria. The final multidisciplinary fall risk evaluation tool therefore includes:

• Previous falls; A positive fall history in the preceding 6 months predicts future falls [13-17,19,26-29,33-35,38,44,51].

• Medication; Number, type and doses of drugs as well as times of intake can influence the risk of falling.

• Locomotor functions; The factors muscle weakness, mobility impairments, disturbed balance, and independency of transfers, which all can increase the risk of falling, were taken together as one item called locomotor functions.

• Assistive and protective devices; For the assistive and protective devices, both the choice and the use of them have to be correct in order to create safe conditions for the patient because wrong choice and/or use enhances the fall risk.

### Second consultation of experts: practical use of the tool

Eight experts responded to the (e-mail) questionnaire. A majority agreed on involving different disciplines in the fall risk evaluation tool, stressing the multidisciplinary aspects of the tool and the importance incorporating the tool in a cyclic procedure: fall risk evaluation at admission; an evaluation after a fall accident; an evaluation at request of the ward; and a periodical repetition of the tool two times a year. Based on the answers of the experts, the members of the research team developed practical guidelines regarding the use of the multidisciplinary fall risk evaluation tool on the psychogeriatric wards. These guidelines are presented in table 3, and described in more detail below.

#### Previous falls

A fall is defined as an event which results in a person coming to rest advertently on the ground or other level (adjusted version of the definition of the Kellog International Work Group) [52]. At admission to the nursing home, information with regard to the fall history in the previous 6 months has to be gathered from the general practitioner, family members and if possible from the patients themselves. Because information about the fall history is gathered retrospectively it is important to use more than one source of information, whenever possible. Obviously the self-report of falls among demented persons may be very unreliable due to their cognitive problems.

The information that has to be gathered includes the number of fall accidents, the possible causes and circumstances of the fall, the consequences of the fall, and the preventive actions already taken [53]. Although obviously fall history it self cannot be influenced, the analysis of the fall history allows the nursing home team to assess whether the factors which caused the falls in this specific patient can be influenced. The more we know about the fall history, the better we can anticipate upon the fall related causes and circumstances. The fall history allows the team also to evaluate the fall preventive policy with regard to individual patients.

#### Medication

The medication used by the patient has to be registered by the nursing home physician who will consequently assess its influence on fall risk. The number of drugs as well as the type, doses and time of intake must be registered. The combination of four or more drugs enhances the risk of falling. Some drugs, particularly cardiovascular and psychotropic drugs, the latter often used in dementia patients, are known to have possible side effects such as drowsiness, dizziness, unstable blood pressure and confusion and thereby can enhance fall risk [13,15-17,19,21,23,26-29,32,34,38,44,45,51].

Regular monitoring of the resident's medication is important because in most nursing home patients, including the demented, we observe polypharmacy, with all possible negative implications.

#### Locomotor functions

Clinical judgement of the patients' mobility by the multidisciplinary team occurs in every day routine and gives important information about possible problems with standing, walking, activities of daily living and transfers and with regard to wandering behaviour, restlessness during the night and other risky behaviour [2,6,13-29,31-33,35-39,41,44,50,51]. In addition to this clinical judgment the Barthel score and the Tinetti test (POMA) are valid screening tools, enabling us to measure more specifically the activities of daily living and the functioning of the mobility apparatus [55]. The Tinetti test assesses both the balance (9 items) and the gait (6 items). The maximum score is 28 points. The scores 19–24 indicate an increased risk of falling. The scores below 19 indicate great risk of falling. The Tinetti test has important practical use: the scores tell us either to focus on balance, on gait or on both. Nurses can observe the general, functional locomotor functions during daily activities; the more specific tests can be performed by an occupational therapist or a physiotherapist.

#### Assistive and protective devices

Taking into account the physical abilities and mental status of the patients involved, the evaluation of both correct choice and use of assistive (walking aids, transfer aids) and protective (external hipprotector, alarmcushions/sensors) devices, can be performed by the clinical judgment of nurses, physiotherapists and occupational therapists [2,14,30,31,47].

#### Tailoring interventions based on the fall risk evaluation

Based on the results of the fall risk evaluation the nursing home staff using the tool has to decide what specific fall preventive interventions are needed for each individual patient. These interventions could include:

• Anticipating upon the causes and circumstances of the fall;

• Critically reviewing and monitoring medication intake (type, number, dose and time of intake);

• Offering exercise programmes specifically targeted at the needs of the individual patient;

• Carefully reassessing the need for assistive and protective devices, and promoting the correct use of these devices.

## Discussion

The aim of this study was to develop a feasible and evidence based multidisciplinary fall risk evaluation tool for multidisciplinary teams in wards for demented nursing home patients. The tool evaluates five important fall risk factors in demented elderly: *previous falls, use of medication, locomotor functions*, and *(correct) choice and use of assistive and protective devices*. The disciplines that may be involved in using this tool are nursing home physicians, nurses, occupational therapists and physiotherapists. It is important to incorporate the multidisciplinary fall risk evaluation tool in a cyclic monitoring procedure. The results of executing this tool target the multidisciplinary and multifactorial fall preventive actions tailored for each individual patient.

### Methodological considerations

A strong aspect of this multidisciplinary fall risk evaluation tool is that it is both evidence and practice based. This has resulted in a tool, which is supported by evidence in scientific literature, and also seems to fit easily into the daily nursing home routine. The fact that we partly based the tool on opinions of experts, however, may at the same time be considered as a weak point of the study because the results obviously depend on the specific composition of the expert group. Despite that, in our opinion the group contributing to the development of the tool was well balanced and representative for the available Dutch expertise on falls in the community as well as in institutions for chronic care.

Obviously, performing this multidisciplinary fall risk evaluation tool in demented nursing home patients does not guarantee that all risk factors for falls in an individual patient will be detected. Therefore it remains very important that sufficient attention is paid to interventions with regard to other factors that in the opinion of nursing home physicians, nurses, and/or paramedical staff can contribute to falls in individual patients.

### Practical implications

At this moment most nursing homes in the Netherlands do not have specific guidelines or structured programmes for the prevention of fall incidents among demented patients [1]. In developing such guidelines the multidisciplinary fall risk evaluation tool, as presented here, involves the first step of an effective fall preventive programme. In addition, complete and integral fall preventive programmes should also include possible interventions that may be targeted by the results of using this evaluation tool. Of course, these programmes also should provide information about general fall preventive measures, for instance, educational programs for the nursing home team, and realisation of a safe nursing home environment [5,57].

Currently we are performing a randomised controlled trial among demented nursing home patients in the Netherlands in which we are testing the effectiveness of the fall risk evaluation tool and the multifactorial interventions specifically targeted by this tool [58]. The trial is accompanied by an evaluation of the feasibility and acceptability of the fall risk evaluation tool, in order to further optimise it and to make it more suitable for use in daily practice.

## Conclusion

This evidence and practice based multidisciplinary fall risk evaluation tool can form the basis for a multifactorial and multidisciplinary intervention aimed to prevent falls and their negative consequences in demented nursing home patients.

## Competing interests

The author(s) declare that they have no competing interests.

## Authors' contributions

All authors read and approved the final version of the manuscript. All authors contributed to the critical evaluation of the writing.

NJCL carried out the study and drafted the manuscript.

DBPJ contributed to the acquisition of data, revised the manuscript and supervised the methodological aspects.

HJCM helped to draft the manuscript, revised the manuscript, and supervised the methodological aspects.

WLP supervised the study and revised the manuscript.

HWJA has contributed to the interpretation of data and revised the manuscript.

CHFJM has contributed to the interpretation of data and revised the manuscript.

SJMGA helped to draft the manuscript, revised the manuscript, and supervised the process of writing.

## Pre-publication history

The pre-publication history for this paper can be accessed here:



## Figures and Tables

**Table 1:** List of risk factors for falls derived from literature.

high age [13,14,16,17,32,37] female gender [14,32,44] weight (e.g. low BMI) [38] previous falls [13-17,19,26-29,33-35,38,44,51] dependency in activities of daily life [14,17,19,26,31,35-38,51] acute disease(s) [2,38] chronic disease(s) [43] neurological diseases [19,32,44] cardiopathy (e.g. heart failure) [20,42,44,45] hart rate (e.g. arrhythmia) [2] high blood pressure [2,26] orthostatic hypotension [12,19] medication (e.g. psychotropic, sedative, neuroleptic, antidepressive) [13,15,16,17,19,21,23,26-29,32,34,35,38,44,45,51] polypharmacy [32,35] alcohol abuse [37] pain [2] dizziness [19,37] diabetes mellitus [13]	urine incontinence [38,41] cognitive problems [13,19,35,40,45,51] confusion [30,42,44,45] depression [33] fear of falling [34] behavioural problems [15,17] disturbed vision [20,38,39,42,44] hearing problems [42,44] independency of transfers [14,16] disturbed balance [13,14,17,19-22] bad quality of co-ordination [6,38] mobility impairments [13,14,17-29,32,33,35-37,39,41,44,51] muscle weakness [20] arthrosis [32] gait disorders [2,26-29,50] wandering [14] foot defects [35] inappropriate footwear [46] environmental factors [2,6,26,33,47-49,51] assistive devices [14,30,31] protective devices [2,47]

**Table 2:** Risk factors for falls related to the inclusion criteria.

**Risk factors for falls**	**1**	**2**	**3**	**4**	**5**	**6**	**All**
Previous falls	+	+	+	+	+	+	+
Chronic disease(s)	+	-	+/-	+/-	+/-	-	-
Medication	+	+	+	+	+	+	+
Disturbed vision	+	+	+/-	+/-	+/-	+	-
Independency of transfers	+	+	+	+	+	+	+
Disturbed balance	+	+	+	+	+	+	+
Bad quality of co-ordination	+	+	+	+	+	-	-
Mobility impairments	+	+	+	+	+	+	+
Muscle weakness	+	+	+	+	+	+	+
Foot defects	+	+	+	+	+	-	-
Assistive devices	+	+	+	+	+	+	+
Protective devices	+	+	+	+	+	+	+

1 = Expert scores ≥ 6

2 = Literature addressing nursing homes

3 = Evaluation possible in nursing homes

4 = Factor can be modified

5 = Intervention applicable in nursing homes

6 = OR/RR ≥ 1.5

All = Fulfilling all inclusion criteria

+ = criterion fulfilled; +/- = ambiguous; - = criterion not fulfilled

**Table 3:** Practical use of the multidisciplinary fall risk evaluation tool for demented nursing home patients.

**WHAT?**	**HOW?**	**WHO?**	**WHEN?**
Previous falls	data from GP (hetero) anamnesis fall registration [52]	nursing home physician nurses/carers	
Medication	medication list	nursing home physician	At admission After a fall incident At request ward Periodical repetition (2 × per year)
Locomotor functions	Barthel index [30] Tinetti (POMA) [55]	nurses/carers occupational therapist physiotherapist	
Assistive and protective devices	Check-list concerning - choice - use	nurses/carers occupational therapist physiotherapist	
